# An Exploratory Study of a Novel Combined Therapeutic Modality for Obsessive-Compulsive Disorder

**DOI:** 10.3390/brainsci12101309

**Published:** 2022-09-28

**Authors:** Yueqi Huang, Hangyi Yang, Cheng Zhu, Xiaoying Jiang, Wenjing Zhu, Yan Liang, Lisha Ma, Yunzan Wang, Wenxin Tang

**Affiliations:** 1 Department of Psychiatry, Affiliated Mental Health Centre and Hangzhou Seventh People’s Hospital, Zhejiang University School of Medicine, Hangzhou 310013, China; 2 Fourth Clinical School, Zhejiang Chinese Medical University, Hangzhou 310013, China

**Keywords:** obsessive–compulsive disorder, combined therapeutic modality, cognitive behavioral therapy, repetitive transcranial magnetic stimulation, insight

## Abstract

Objective: To explore whether a systematic combined therapeutic modality (CTM) could quickly and effectively improve the severity of obsessive–compulsive disorder (OCD) and the insight of OCD patients. Methods: Included in this study were 100 patients with OCD according to the 5th Edition of the Diagnostic and Statistical Manual of Mental Disorders (DSM-5), for a 2-week short-term treatment. They were assigned to a drug-alone group (*n* = 57), and a CTM group (*n* = 43) using drug treatment in combination with cognitive behavioral treatment (CBT) and repetitive transcranial magnetic stimulation (rTMS). The therapeutic outcome was assessed by the Yale–Brown Obsessive–Compulsive Scale (Y-BOCS), Brown Assessment of Beliefs Scale (BABS), 24-item Hamilton Depression Scale (HAMD-24) and Hamilton Anxiety Scale (HAMA) before and after treatment. All data were treated with SPSS25.0 Software. Results: After the 2-week treatment, the success rate in the CTM group was significantly higher than that in the drug-alone group. Y-BOCS overall and factor scores were decreased as compared with those before treatment in both groups. HAMD, HAMA and BABS overall scores were all decreased after treatment in the CTM group. In addition, compared with the drug-alone group, the Y-BOCS overall score and factor score, HAMD overall score and HAMA overall score were all decreased significantly in CTM group, while the Y-BOCS score reduction rate was increased significantly. Insight was improved in eight cases (57.14%) in the CTM group containing 14 cases with poor insight. Multinomial logistic regression analysis showed that CTM was beneficial for the insight improvement of OCD patients (OR = 91.04–139.68); this improvement was more pronounced in patients with low baseline BABS overall scores (OR = 0.07). Conclusion: CTM may be an effective short-term strategy to improve the severity of OCD and insight of OCD patients and, therefore, is worthy of clinical promotion and application.

## 1. Introduction

Obsessive–compulsive disorder (OCD) is a chronic disabling disease with an unsatisfactory response to either medical or psychological therapy. The global lifetime prevalence of OCD is 0.8–3% [1]. The latest epidemiological study shows that the lifetime prevalence of OCD in Mainland China is about 2.4% [2]. The main clinical features of OCD are intrusive thoughts and/or repetitive behaviors, which can be simultaneously accompanied with anxiety, fear and other negative emotions, greatly affecting the daily life and social function of the patients and imposing huge burdens of disease [3,4].

The etiology of OCD is complex, involving social, mental and biological factors, and its pathogenesis remains unclear. Multiple, currently available guidelines recommend selective serotonin reuptake inhibitors (SSRIs) and cognitive behavioral therapy (CBT) containing the component of exposure-response prevention (ERP) as the first-line treatment for OCD [5,6]. Although CBT alone or in combination with SSRIs remains a preferred initial treatment strategy, 40–60% of patients still suffer symptoms of residual damage even after adequate initial treatment [7]. In recent years, OCD treatment has gradually transited to the safer and more fast-acting neuromodulation technique. Based on the understanding about the relationship between OCD and cortico-striato-thalamo-cortical (CSTC) circuitry, different brain neurostimulation techniques have been used to promote the rehabilitation of OCD patients by acting on the CSTC circuitry, especially patients with refractory OCD [8,9]. Repetitive transcranial magnetic stimulation (rTMS) is the most common non-invasive brain neurostimulation technique for the treatment of a series of mental and neurological diseases, especially refractory OCD. A meta-analysis reports that rTMS is effective for OCD, and an effective alternative for the treatment of refractory OCD as well [9]. However, the therapeutic effect of rTMS loses its significance 12 weeks after treatment [10]. Given the low action of OCD treatment, limited therapeutic efficacy and increased medical expenditure due to the COVID-19 pandemic in recent years [11], it is imperative to explore a systematic, faster and more effective strategy for the treatment of OCD.

Previous exploration of effective treatments for OCD has demonstrated that drugs, CBT and rTMS are all effective for the treatment of OCD. However, previous studies mainly focused on a singly or pairwise combined therapy for OCD, and few studies have reported triplet combined therapy for OCD (except for refractory OCD). In addition, according to the Diagnostic and Statistical Manual, 5th Edition (DSM-5), OCD patients can have no insight about his/her disease, which expands the diagnostic scope of OCD. Studies have demonstrated that the symptoms and signs of OCD patients with poor insight may be more severe and the therapeutic outcome is often unsatisfactory. As a result, these patients may have more severe symptoms, poorer responses to medical treatment and more severe social function impairment, leading to higher rates of unemployment [12] and imposing socioeconomical burdens on both the family and society [13]. Therefore, increasing the insight of OCD patients is primarily important for improving the limitations of OCD treatment [14]. The aim of the present study was mainly to evaluate the effectiveness of a novel combined therapeutic modality (CTM) using drug treatment in combination with short-term CBT and rTMS for OCD, to see whether this novel modality could improve the insight of OCD patients, and explore the main factors affecting the insight of OCD patients.

## 2. Patients and Methods

### 2.1. Patients

Included in this study were 100 outpatients and inpatients with OCD who sought medical treatment in the Mental Health Center of Zhejiang Medical University (Hangzhou, China) between January 2020 and April 2022. Of them, 56 were outpatients who were assigned to the drug-alone group, and 44 were inpatients who were assigned to the CTM group. The inclusion criteria were: (1) the diagnosis of OCD according to the 5th Edition of the Diagnostic and Statistical Manual of Mental Disorders (DSM-5); (2) Yale–Brown Obsessive–Compulsive Scale (Y-BOCS) score ≥16; (3) patients of Han ethnicity aged 16–65 years; (4) no history of receiving psychological treatment for OCD in the past month; and (5) a secondary school or higher level of education. The exclusion criteria were: (1) refractory OCD: The definition of refractory OCD is that the patient has no response at the maximum tolerated doses for the sufficient treatment course. The treatment course includes a minimum of three attempts of SSRIs (must include the clomipramine), a combination of two different second-generation antipsychotics (SGA), or a minimum period of 3 months of CBT; (2) patients with severe somatic diseases; (3) a history of using psychoactive substances; (4) patients with severe suicide tendency with the Mini International Neuropsychiatric Interview (MINI) score >9; (5) pregnant or lactating women; and (6) consistent with other diagnoses in DSM-5. This study was approved by the Ethics Committee of the Mental Health Center of Zhejiang Medical University, and written informed consent was obtained from the participating patients or their guardians before initiation of the study.

### 2.2. Assessment Tools and Methods

The diagnosis of the included patients was confirmed by the attending or higher-ranking psychiatrists and verified by the Chinese version of MINI for checkup diagnosis to exclude comorbidities. Before initiation of the study, all members of the research team received training about the research program and Y-BOCS consistency training. The symptoms of OCD were assessed by Y-BOCS; insight was assessed by Brown Assessment of Beliefs Scale; the state of depression was assessed by HAMD-24; and the state of anxiety was assessed by HAMA.

### 2.3. General Data Collection

The demographic data and basic clinical characteristics of the eligible patients were collected by using the self-made general information form, including sex, ethnicity, age, age at the time of first onset of the symptoms, the general course of disease, educational level, family history, and somatic commodities.

### 2.4. Assessment of Severity of the OCD Symptoms

The severity of OCD symptoms was assessed by Y-BOCS, knowing that it is an examiner-rating scale (ERS) consisting of 10 items using a 0–4 scoring system. The overall score of the scale is the sum of the four items, and the higher the score, the more severe the symptoms. As the scale has a good reliability and validity, it has been widely used in clinical practice [15].

### 2.5. Insight Assessment

By virtue of its good reliability and validity, the Chinese version of BABS is commonly used for assessing the insight of OCD patients in China [16]. Poor insight is indicated by an overall BABS score of ≥12 (mean score of 2 for each BABS item) and ≥3 for the conviction item (fairly or completely convinced that belief/worry is true) [17].

### 2.6. Outcome Measure

The patient assessment was performed at the baseline time and by the end of 2-week treatment using Y-BOCS, HAMD-24, HAMA and BABS. The second assessment of the outpatients was conducted either in person or through telephonic interview.

The primary outcome measure was the Y-BOCS score reduction rate of both groups at the end of 2-week treatment using the following equation: Y-BOCS score reduction rate = (pre-treatment score–score at the end of 2-week treatment)/pre-treatment score * 100%. Successful treatment was defined as Y-BOCS score reduction rate ≥30%. The secondary outcome measure included the HAMD-24, HAMA and BABS scores at the end of 2-week treatment in both groups.

### 2.7. Patient Treatment

CTM included 2-week drug therapy in combination with short-term CBT and rTMS in the included inpatients, consisting of 3 sessions of group psychological therapy per week (90 min per session), totaling 6 sessions, 5 individual CBT sessions per week (60–90 min per session, including exposure and reaction prevention) totaling 10 sessions, 5 rTMS stimulations per week, totaling 10 simulations, and medical treatment (SSRIs/clomipramine and other synergists (aripiprazole and risperidone). The content of CBT each week was according to the results of evidence-based interviews and standardized session-by-session protocols, including standardized exercise and home assignment forms. Before treatment, each patient would be interviewed personally for about 90 min to learn about their conditions, assess the severity of symptoms, provide disease and health education, and establish a good and mutually reliable doctor–patient relationship. At the same time, the patient would be informed of the methods and formalities of group therapy and individual therapy. The initial phase mainly included self-introduction, theoretical fundamentals about event-related potential (ERP) and CBT, home assignments, and formulation of the exposure list. The intermediate phase mainly included perfecting the exposure list through negotiation with the patient to effect gradual exposure based on preventing the occurrence of compulsive behavior or avoidance behavior. Only when the Subjective Units of Distress Scale (SUDs) score was ≤30 could the exposure exercise be proceeded to the next item. The home assignment should include at least one challenge per day and each challenge should last one hour. During the period of treatment, misrecognition and irrational beliefs should be corrected and compulsive behaviors should be stopped. In addition, mindfulness-based cognitive therapy was applied to treat obsessions and reconstruct cognition (to reconstruct cognition, we needed to check whether mindfulness cognitive behavioral therapy had this function). The last phase mainly included consolidating the changes that the patient had made, deeply analyzing and correcting the patient’s misrecognition (deep analysis should be the content of the intermediate phase), and discussing and learning behavioral strategies of how to prevent disease recurrence. rTMS therapy was mainly located at the left prefrontal lobe at 10 Hz with 60 pulse trains and 40 per burst at a 15 s interval and 2400 pulses at a time. As there is no significant difference in the therapeutic effect on OCD between different SSRIs [18], all patients continued with the use of one kind of SSRIs or in combination with the SGA as synergists (aripiprazole and risperidone).

### 2.8. Data Treatment

All statistical analyses were performed by SPSS25.0. Normality of measurement data was verified by single sample Kolmogorov–Smirnov (K-S) test, and those of normal distribution were expressed as the mean ± standard deviation (SD) and tested by t test with two independent samples or paired samples. Measurement data of abnormal distribution were expressed as [median (minimal value and maximal value)]. Continuity variables and classification variables were analyzed by descriptive analysis (nonparametric rank sum test) and chi-square test ($\chi^{2}$ test). If the theoretical frequency of the sample was less than 5, Fisher exact test was used for comparison between groups. After treatment, the patients were classified into three groups—a poor insight group, and an improved insight group (insight improved from poor insight to good insight after treatment), and a good insight group. A multinomial logistic regression analysis was performed, the BABS overall score in baseline and treatment modality were the independent variables. In addition, pseudo R-square was determined by Cox–Snell and Nagelkerke methods. Two-tailed hypothesis test was conducted at α = 0.05.

## 3. Results

### 3.1. General Clinical Features

At baseline, there was no significant difference in age, sex, family history, age at the time of initial symptom onset, course of disease, baseline Y-BOCS overall score, baseline Y-BOCS obsessions score, baseline Y-BOCS compulsions score, baseline HAMD overall scorebaseline HAMA overall score, and numbers of poor insight between the two groups. However, patients in the CTM group had even lower educational levels(*z* = −3.656), higher BABS scores (*z* = −2.220) representing poorer insights than those in the drug-alone group (both *p* < 0.05) (Table 1).

### 3.2. Therapeutic Outcome Assessment and Comparison of Y-BOCS, HAMD, HAMA and BABS Overall Scores before and after Treatment between the Two Groups

After the 2-week treatment, seven patients (12.5%) in the drug-alone group achieved effectiveness versus 23 patients (52.3%) in the CTM group, showing a significant difference between the two groups ($\chi^{2}$ = 18.561, *p* < 0.05) (Table 1). There was no significant difference in baseline Y-BOCS overall score (*z* = −0.254), factor score (obsessions: *z* = −0.551, compulsions: *z* = −0.439), HAMD overall score (*z* = −0.589) and HAMA overall score(*z* = −0.212) between the two groups (*p* > 0.05), but there was a significant difference in baseline BABS overall score between the two groups (*z* = −2.220, *p* < 0.05). After the 2-week treatment, Y-BOCS overall score (drug-alone: *t* = 6.817, CTM: *t* = 11.880) and factor score (drug-alone: obsessions *t*= 7.155, compulsions *t* = 5.906) (CTM: obsessions *t* = 11.085, compulsions *t* = 10.475) were decreased as compared with those before treatment in both groups. After treatment, HAMD overall score (*t* = 6.492), HAMA overall score (*t* = 7.243) and BABS overall score (*t* = 8.852) in CTM group were all decreased as compared with those before treatment. In addition, after treatment, Y-BOCS overall score (*z* = −4.106), factor score (obsessions: *z* = −3.586, compulsions: *z* = −4.021), HAMD overall score (*z* = −3.078) and HAMA overall score(*z* = −3.146) in CTM group were all significantly lower than those in drug-alone group, and Y-BOCS score reduction rate (*z* = −4.873) was significantly higher than that in drug-alone group (all *p* < 0.05). After treatment, the severity of OCD (including obsessions and compulsions), depression and anxiety, and insight were all improved in CTM group, while only the severity of OCD (including obsessions and compulsions) and insight were improved in the drug-alone group (Table 2).

### 3.3. Correlation between Treatment Response-Related Variables and Insight after Treatment

After the 2-week treatment, there were 18 cases of poor insight, 9 cases of improved insight, and 73 cases of good insight, in total. Finally, there was one case (5.6%) of poor insight, 7 cases (77.8%) of improved insight, and 22 cases (30.1%) of good insight, showing a significant difference in the effective rate between the three groups ($\chi^{2}$ = 14.905), and comparison between the three groups also showed a significant difference (*p* < 0.05). The Y-BOCS score reduction rate in the improved insight group and good insight group was significantly higher than that in poor insight group (48.72 ± 19.52% and 25.51 ± 19.27% versus 12.13 ± 18.33%, *z* = 20.171, *p* < 0.05). The therapeutic effect in the former two groups was also better than that in the poor insight group, especially in the improved insight group ($\chi^{2}$ = 14.905). However, there was no significant difference in SGA between the three groups ($\chi^{2}$ = 2.259) (Table 3).

### 3.4. Result Analysis by Multinomial Logistic Regression

After treatment, insight was improved significantly in nine cases including eight cases in CTM, reaching the diagnostic criteria of good insight (improved insight group), and no significant improvement in insight was observed in 18 cases, which were still constituent with the diagnosis of poor insight. Compared with the poor insight group, CTM could help improve the patient’s insight, especially in patients with low baseline BABS overall scores. The likelihood ratio of CTM was $\chi^{2}$ = 114.43 and *p* < 0.01, showing statistical significance. The pseudo R [2] detected by the Cox-Snell method and Nagelkerke method was 0.682 and 0.758, respectively, indicating the modality had a good fitting (Table 4). The regressive equation was obtained as follows: log(*P*_improved insight_/1−*P*_improved insight_) = 7.383 − 0.770·BABS_overall score in baseline_ + 4.511·CTM treatment; log(*P*_good insight_/1−*P*_good insight_)= 30.136 − 2.630·BABS_overall score in baseline_ + 4.939·CTM treatment.

## 4. Discussion

The prevalence of OCD is high, mainly characterized by obsessions and compulsive compulsions as represented by severe time consumption and painful experience, which greatly reduces the quality of life (QoL) of the patients. This QoL reduction is similar to that seen in schizophrenic patients [19], thus imposing huge economic burdens on both patients and society [3,20]. The guidelines for OCD prevention and treatment recommend SSRIs and CBT as the first-line treatment for OCD at present, and rTMS as the synergistic therapy for refractory OCD [7]. CBT is generally recognized as an evidence-based effective treatment for OCD, but its clinical application is limited in China because of the time-consuming procedure and shortage of professionals with the OCD specialty [21]. As the pathological mechanism of medical treatment for OCD remains unclear and there is inadequate basic research on OCD, there are not many specific drugs available for OCD. In addition, the existing drugs work slowly with a low effective rate. rTMS is effective for refractory OCD but is time-limited [10]. Given the current situation of OCD treatment, it is imperative to seek a fast and effective new CTM for OCD.

The results of the present study showed that CTM could relieve the symptoms (including obsessions and compulsive compulsions) of OCD patients quickly and effectively, with an effective rate of 52.3% after a 2-week treatment. Previous studies showed that SSRIs could effectively attenuate the severity of OCD symptoms in the acute phase, and continued therapy could further relieve the symptoms and reduce the risk of disease recurrence [6]. However, even after adequate dosage and duration treatment, there were still 40–60% OCD patients who had poor or no symptomatic improvement, and only a small number of patients could achieve complete remission of the symptoms [18]. For patients with refractory OCD, the effective rate of SGA administration as synergists (aripiprazole and risperidone) is about 40–55% [22]. It was found in our study that CTM could improve the obsessions and compulsions of OCD patients, though the effective rate was only 12.5%. This may be because of the slow onset of the drugs, and the optimal therapeutic effect could be observed only after treatment with the maximum tolerated dose for 8–12 weeks [23]. However, the present study only lasted two weeks. Despite the short duration of treatment, the effective rate in our CTM group was high, at 52.3%, indicating that the CTM is a fast and effective strategy for the treatment of OCD. Some studies pointed out that CBT should be the most effective first-line treatment of OCD as long as the conditions permit [7]. Either individual CBT or group CBT can improve the compulsive symptoms effectively [24]. In the present study, the patients in combined treatment groups received 6 sessions of group CBT and 10 sessions of individual CBT, which is one of the reasons why the compulsive symptoms could be improved remarkably. In addition, rTMS is a non-invasive neuromodulation technique by modulating the dysfunctional brain zone, especially the activity of the CSTC circuitry [9]. It has been approved by the regulatory authorities for the treatment of refractory OCD [25]. A study reported that rTMS could be used safely and effectively as an early synergistic strategy for OCD treatment [26]. In the present study, we localized rTMS at the dorsolateral prefrontal cortex (DLPFC), one of the important brain zones of CSTC circuitry. Application of rTMs, especially high-frequency rTMS in DLPFC can produce an obvious therapeutic effect on OCD [25,27,28]. This also provides powerful evidence to support the therapeutic efficacy of CTM. The results of the present study also demonstrated that either medical therapy, CBT or rTMS could improve the symptoms of depression and anxiety of OCD patients [27,29].

Insight refers to the awareness of the individual that his or her obsessions or compulsion is a symptom of OCD; it is not an actual existing and natural belief or behavior with any protective meaning. Insight has long been regarded as an important clinical feature of OCD [30].

Poor insight occurs in 13–36% of OCD patients [31]. The rate of reduction in Y-BOCS score is relatively low and the therapeutic outcome is relatively poor in OCD patients with poor insight even after systematic treatment [32,33]. It was found in our study that insight was improved significantly to a level consistent with the diagnostic criteria of good insight in nine OCD patients with poor insight. Of the nine patients, eight (88.9%) were inpatients receiving CTM. In addition, the therapeutic outcome in patients with poor insight was usually unsatisfactory, and only one patient (5.6%) achieved the effective rate. The therapeutic outcome was best in patients who achieved improved insight. These results demonstrate that the CTM described in this study could improve the patient’s insight, and that insight improvement was associated with an improved therapeutic outcome.

To further explore factors affecting insight improvement, we performed a multinomial logistic regression analysis and the results showed that CTM (medical therapy in combination with CBT and rTMS at DLPFC) could better improve insight as compared with drugs alone, and this improvement was more pronounced in patients with low baseline BABS overall scores. Other studies also reported that CBT and rTMs could help improve the insight of OCD patients [33,34]. In addition, insight improvement may be related to some particular brain zones such as DLPFC [26]. DLPFC is a significant node of the frontal cognitive circuit which is involved in executive control and cognitive processes [35]. After CBT, DLPFC activity was enhanced (within the normal range), and the connection between the DLPFC and cerebellum was also strengthened. In addition, different rTMS protocols have shown significant improvements in cognitive functions such as the excitatory effects of intermittent theta burst stimulation (TBS) [36,37]. A further study demonstrated that high-frequency rTMS could excite the nerve by stimulating DLPFC [38], increase the integrity of the prefrontal white matter, and improve its dysfunction [39]. Bidet-Caulet et al. pointed out that the prefrontal lobe was closely related to cognitive function [40]. Therefore, we can further postulate that CBT and repeated high-frequency rTMS at the prefrontal lobe could modulate cortical excitement, activeness and plasticity [41], which induces the changes of Dopamine and other neurotransmitters [42], leading to changes in brain functional connection and microstructural change of the white matter [43,44] and finally participating in improving the insight of OCD patients. This may be the mechanism of CTM in improving the insight of OCD patients, though further study is required to verify our postulation.

This study has some limitations. First, the sample size was relatively small, and therefore larger sample-size studies are required to confirm the therapeutic outcome of CTM of the present study. Second, the follow-up duration was relatively short and therefore it is difficult to forecast its long-term outcome. Finally, CBT consumes a great deal of time, which may affect compliance to it, especially in the context of the COVID-19 epidemic environment. As a result, the target audience was limited. The evidence demonstrated that the therapeutic effectiveness of remote CBT (through the online platform) was similar to that of face-to-face therapy [45], and further exploration is warranted to standardize network CBT for the sake of creating more benefit to more OCD patients.

Considering the limitations above, further exploratory research may need to be undertaken. In summary, the results of the present study have demonstrated that the CTM of using drug therapy in combination with CBT and rTMS described in this study may be a fast and effective short-term strategy for the treatment of OCD patients in that it can improve their symptoms, insights and therapeutic outcomes, thus promoting their early return to society. This novel CTM is worthy of clinical promotion and application.

## Figures and Tables

**Table 1:** General clinical features and scale between drug-alone group and CTM group.

		Drug-Alone Group (56)	CTM Group (44)	*χ*^2^/*z*	*p*
Age (years)		26.5 (18.46)	24 (16.60)	−0.699	0.486
Sex	female	29	23	0.002	0.961
	male	27	21		
Educational levels (years)		15 (6.21)	12 (1.19)	−3.656	<0.001
Family history no		50	39	0.000	1.000
yes		6	5		
Age at onset of OCD, in years		21 (7.45)	18.5 (9.44)	−0.622	0.534
Duration of illness, in months		64 (1.240)	57 (2.500)	−0.431	0.667
Y-BOCS (baseline) overall		25.64 ± 2.98	25.00 ± 5.75	−0.254	0.799 *
Obsessions		13 (10.17)	13 (6.19)	−0.551	0.582
Compulsions		13 (9.16)	13 (0.19)	−0.439	0.661
HAMD (baseline)		10 (0.35)	11.5 (1.39)	−0.589	0.556
HAMA (baseline)		7 (1.28)	8 (0.32)	−0.212	0.832
BABS (baseline)		7 (2.21)	8.5 (2.18)	−2.220	0.026
Insight, *n*(%) poor		13 (23.2)	14 (31.8)	0.925	0.336
good		43 (76.8)	30 (68.2)		
Treatment response, effectiveness *n*(%)		7 (12.5)	23 (52.3)	18.561	<0.001

CTM = combined therapeutic modality; OCD = obsessive–compulsive disorder; Y-BOCS = Yale–Brown Obsessive–Compulsive Scale; HAMD-24 = 24-Item Hamilton Depression Scale; HAMA = Hamilton Anxiety Scale; BABS= Brown Assessment of Beliefs Scale; * represents the variance is not uniform using nonparametric rank sum test.

**Table 2:** Therapeutic outcome assessment and comparison of Y-BOCS, HAMD, HAMA and BABS overall scores before and after treatment between the two groups.

	Baseline	2-Weeks	*t/z*	*p*
Y-BOCS overall score				
drug-alone group	25.64 ± 2.98	21.36 ± 4.91	6.817	<0.001
CTM group	25.00 ± 5.75	16.14 ± 6.92 *	11.880	<0.001
Y-BOCS obsessions				
drug-alone group	12.80 ± 1.72	10.79 ± 2.63	7.155	<0.001
CTM group	12.91 ± 3.83	7.80 ± 4.08 *	11.085	<0.001
Y-BOCS compulsions				
drug-alone group	12.84 ± 1.88	10.80 ± 2.53	5.906	<0.001
CTM group	12.11 ± 3.83	7.8 ± 4.08 *	10.475	<0.001
HAMD overall score				
drug-alone group	14.31 ± 10.36	12.41 ± 11.71	1.529	0.132
CTM group	13.25 ± 10.77	5.98 ± 7.33 *	6.492	<0.001
HAMA overall score				
drug-alone group	9.04 ± 6.08	7.91 ± 7.64	1.448	0.153
CTM group	10.00 ± 8.68	4.23 ± 5.42 *	7.243	<0.001
BABS overall score				
drug-alone group	7.77 ± 3.95	7.21 ± 3.52	4.111	<0.001
CTM group	9.66 ± 4.47 *	7.41 ± 4.08	8.852	<0.001
Rate of reduction in Y-BOCS overall score				
drug-alone group		10.53 (4.77)	−4.873	<0.001
CTM group		36.17 (3.73)		

CTM = combined therapeutic modality; Y-BOCS = Yale–Brown Obsessive–Compulsive Scale; HAMD-24 = 24-Item Hamilton Depression Scale; HAMA = Hamilton Anxiety Scale; BABS= Brown Assessment of Beliefs Scale; * represents statistic difference between the drug-alone group and CTM group.

**Table 3:** Correlation between treatment response-related variables and insight after treatment.

	Poor Insight	Improved Insight	Good Insight	*χ*^2^/*z*	*p*
Augmentation with antipsychotics, *n* (%)	5 (27.8)	4 (44.4)	16 (21.9)	2.259	0.323
Rate of reduction in Y-BOCS overall score after treatment	67.8 (0.77)	46.2 (20.711)	23.1 (−4.73)	20.171	<0.001
Status at last assessment				14.905	0.001
Effectiveness, *n* (%)	1 (5.6)	7 (77.8)	22 (30.1)		
Non-effectiveness, *n*(%)	17 (94.4)	2 (22.2)	51 (69.9)		

Y-BOCS = Yale–Brown Obsessive–Compulsive Scale.

**Table 4:** Results analysis by multinomial logistic regression.

Variables		B (SE)	OR	95% CI	Wald	*p*
I	CTM treatment	4.51 (1.64)	91.04	3.65–2273.00	7.55	0.006
II	CTM treatment	4.94 (2.01)	139.68	2.72–7186.66	6.04	0.014
	BABS overall score in baseline	–2.63 (0.83)	0.07	0.01–0.36	10.15	0.001

I represents from poor insight in baseline change to good insight after treatment; II represents good insight; CTM = combined therapeutic modality; BABS = Brown Assessment of Beliefs Scale.

## Data Availability

The data supporting the presented results are available on request from the corresponding author. The data are not publicly available to protect the privacy of the patients.
